# Knockdown of CDC20 promotes adipogenesis of bone marrow-derived stem cells by modulating *β*-catenin

**DOI:** 10.1186/s13287-022-03062-0

**Published:** 2022-09-02

**Authors:** Yangge Du, Yunsong Liu, Yongsheng Zhou, Ping Zhang

**Affiliations:** Department of Prosthodontics, National Engineering Research Center of Oral Biomaterials and Digital Medical Devices, National Clinical Research Center for Oral Diseases, Beijing Key Laboratory of Digital Stomatology, Peking University School and Hospital of Stomatology, 22 Zhongguancun South Avenue, Haidian District, Beijing, 100081 People’s Republic of China

**Keywords:** CDC20, BMSCs, Adipogenesis, Bone marrow, *β*-catenin

## Abstract

**Background:**

Bone is a rigid organ that provides physical protection and support to vital organs of the body. Bone loss disorders are commonly associated with increased bone marrow adipose tissue. Bone marrow mesenchymal stromal/stem cells (BMSCs) are multipotent progenitors that can differentiate into osteoblasts, adipocytes, and chondrocytes. Cell division cycle 20 (CDC20) is a co-activator of anaphase promoting complex/cyclosome (APC/C), and is required for ubiquitin ligase activity. Our previous study showed that CDC20 promoted the osteogenic commitment of BMSCs and *Cdc20* conditional knockout mice suggested a decline in bone mass. In this study, we found that knockdown of *CDC20* promoted adipogenic differentiation of BMSCs by modulating *β*-catenin, which suggested a link between adipogenesis and osteogenesis.

**Methods:**

Lentivirus containing a *CDC20* shRNA was used for *CDC20* knockdown in human BMSCs (hBMSCs). Primary mouse BMSCs (mBMSCs) were isolated from *Cdc20*^*f/f*^ and *Sp7-Cre;Cdc20*^*f/f*^ mice. Adipogenesis was examined using quantitative real-time reverse transcription PCR (qRT-PCR) and western blotting analysis of adipogenic regulators, Oil Red O staining, and transplantation into nude mice. *CDC20* knockout efficiency was determined through immunochemistry, qRT-PCR, and western blotting of bone marrow. Accumulation of adiposity was measured through histology and staining of bone sections. Exploration of the molecular mechanism was determined through western blotting, Oil Red O staining, and qRT-PCR.

**Results:**

*CDC20* expression in hBMSCs was significantly decreased during adipogenic differentiation. *CDC20* knockdown enhanced hBMSC adipogenic differentiation in vitro. *CDC20*-knockdown hBMSCs showed more adipose tissue-like constructs upon hematoxylin and eosin (H&E) and Oil Red O staining. *Sp7-Cre;Cdc20*^*f/f*^ mice presented increased adipocytes in their bone marrow compared with the control mice. mBMSCs from *Sp7-Cre;Cdc20*^*f/f*^ mice showed upregulated adipogenic differentiation. Knockdown of *CDC20* led to decreased *β*-catenin levels, and a *β*-catenin pathway activator (lithium chloride) abolished the role of CDC20 in BMSC adipogenic differentiation.

**Conclusions:**

Our findings showed that CDC20 knockdown enhanced adipogenesis of hBMSC and mBMSCs adipogenesis in vitro and in vivo. CDC20 regulates both adipogenesis and osteogenesis of BMSCs, and might lead to the development of new therapeutic targets for “fatty bone” and osteoporosis.

**Supplementary Information:**

The online version contains supplementary material available at 10.1186/s13287-022-03062-0.

## Background

Bone is a complex endocrine organ that facilitates structural support and protection of vital organs [1]. The majority of clinical conditions related to bone loss, including osteoporosis, aging, and hypercortisolism, are known to be accompanied by increased bone marrow fat [2–4]. This process involves the proliferation of BMSCs, their commitment to the adipogenic lineage, and their terminal differentiation [5]. BMSCs are multipotent progenitors giving rise to osteoblasts, adipocytes, and chondrocytes under specific stimulation [6]. As bone marrow adipocytes and osteoblasts share common progenitor cells, the lineage commitment of BMSCs must be dynamic [7]. Establishing a better understanding of fat storage and the osteoblast-adipocyte relationship within the bone marrow niche is necessary to elucidate the mechanisms underlying osteoporosis and age-related diseases.

The differentiation of BMSCs into adipocytes is driven by different transcription factors and regulated by various signaling pathways [6]. Extensive analyses have established the genetic cascade of adipogenesis. In response to adipocytic inducers, the transcription factors C/EBP*β*, C/EBPδ and PPAR*γ*1 are rapidly activated in BMSCs and initiate the adipogenic cascade. This process includes elevated expression of two critical transcription factors responsible for adipogenesis, C/EBP*α* and PPAR*γ*2, followed by an increase in downstream genes characterized as mature adipocytes, including fatty acid binding protein 4 (FABP4) and adiponectin [8–13].

Cell division cycle 20 (CDC20) is the co-activator of anaphase-promoting complex/cyclosome (APC/C) [14]. CDC20 activates APC/C and promotes ubiquitin-dependent degradation of substrates [15]. In addition to participating in cell cycle progression, CDC20 was reported to be associated with many categories of cancers [16], brain development [17], and cellular apoptosis [18]. A high-fat diet is reported to lead to alteration of CDC20 level in pulmonary tissue [19]. The gene expression of *CDC20* was downregulated in obese women compared with that in the controls [20]. Thus, it seems that CDC20 correlates with adiposity; however, its role and mechanisms in BMSC adipogenesis remain elusive.

Our previous study revealed that CDC20 is a positive regulator of bone formation and osteogenesis of BMSCs [21]; and this study aimed to investigate its role in the adipogenic differentiation of BMSCs. To further explore whether CDC20 regulates adipogenesis of BMSCs, we used *CDC20* knockdown BMSCs and deleted the *Cdc20* gene in osteoprogenitors using *Sp7-Cre* mice. The results presented that the levels of CDC20 decreased during the adipogenic differentiation of hBMSCs. Inhibition of CDC20 enhanced the adipogenic differentiation of hBMSCs through decreasing *β*-catenin expression. Furthermore, conditional knockout of *Cdc20* increased the number of adipocytes in bone marrow and the adipogenic differentiation of mBMSCs. This study provided new insights into how CDC20 manipulates the adipogenesis of BMSCs. Exploiting the potential of CDC20 in the BMSC differentiation process might become a new method to enhance bone mass as well as combat bone loss states.

## Materials and methods

### Cell culture

hBMSCs (No: 7500; Lots: 6899, 6881, 6890) and human adipose-derived stem cells (hASCs) (No: 7510; Lots: 2447, 8278, 8279) were purchased from ScienCell Research Laboratories (Carlsbad, CA, USA). Primary mBMSCs were separated from mice by flushing the bone marrow from the long bones. The detailed methods have been published previously [22]. BMSCs or ASCs were cultivated at 37 ℃ in 5% CO_2_ conditions. They were cultivated in proliferation medium (PM), comprising penicillin/streptomycin, 10% (v/v) fetal bovine serum (FBS), and α-minimum essential medium (*α*-MEM) or Dulbecco’s modified Eagle’s medium (DMEM). The adipogenic medium (AM) included α-MEM or DMEM added with 10 μM insulin, 100 nM dexamethasone, 200 μM indomecin, 500 μM 3-isobutyl-1-methylxanthine, penicillin/streptomycin, and 10% (v/v) FBS.

### Mice

*Cdc20*^*f/f*^ and *Sp7-Cre;Cdc20*^*f/f*^ mice were constructed by Biocytogen Co., Ltd (Beijing, China). Briefly, we first constructed *Cdc20*^f/f^ mice using CRISPR-Cas9 system, and then mated them with *Sp7-Cre* mice to delete *Cdc20* in osteoprogenitors. The detailed information of conditional knockout mice in the experiments was reported in our previous work [21]. The primers are shown in Table 1.

**Table 1:** Genotyping primers

*Cdc20* 5′loxP-F	CCTAAACTATGTGGAGTTCAAGGCCA	WT:202 bp
*Cdc20* 5′loxP-R	AGGATCTAGGATCTAGGTGACTCCC	Mut:321 bp
*Cdc20* 3′loxP-F	GAAGCAGCTCCTGTCTTGGAGTTGT	WT:405 bp
*Cdc20* 3′loxP-R	CCACAGCCTGGGTGGAATGGATAAA	Mut:490 bp
*Sp7-Cre* WT-F	TACCAGAAGCGACCACTTGAGC	263 bp
*Sp7-Cre* WT-R	CGCCAAGAGAGCCTGGCAAG	263 bp
*Sp7-Cre* Mut-F	TACCAGAAGCGACCACTTGAGC	445 bp
*Sp7-Cre* Mut-R	GCACACAGACAGGAGCATCTTC	445 bp

### Lentivirus infection

Recombinant lentiviruses targeting *CDC20* were purchased in GenePharma Company (Shanghai, China). The lentivirus vectors contained the green fluorescent protein (GFP) gene and a puromycin-resistance gene. hBMSCs and hASCs were infected with lentiviruses using polybrene (5 μg/ml) to enhance the transfection efficiency. Forty-eight hours later, puromycin (1 mg/ml) was utilized to select the infected cells. The oligonucleotide sequences mentioned are presented in Table 2.

**Table 2:** Sequences of RNA and DNA oligonucleotides

	Sense strand/ sense primer (5′-3′)	Antisense strand/ antisense primer (5′-3′)
Primers		
*GAPDH*	AAGGAGTAAGACCCCTGGACCA	GCAACTGTGAGCAGGGGAGATT
*PPARG*	GAGGAGCCTAAGGTAAGGAG	GTCATTTCGTTAAAGGCTGA
*CEBPA*	GGGCCAGGTCACATTTGTAAA	AGTAAGTCACCCCCTTAGGGTAAGA
*FABP4*	CCTTAGATGGGGGTGTCCTGGT	GCCTTTCATGACGCATTCCACC
*CDC20*	TGTGTGGCCTAGTGCTCCTG	ACACCATGCTACGGCCTTGA
*Gapdh*	CAGGAGAGTGTTTCCTCGTCC	TGAAGGGGTCGTTGATGGCA
*Cdc20*	CTCAAAGGACACACAGCACGG	CGCCACAACCGTAGAGTCTCA
*Pparg*	ACGGGCTGAGGAGAAGTCAC	TCACCGCTTCTTTCAAATCTTGTCT
*Cebpa*	ATTCCTGCTTCCTCTCGGGC	ACTGCCGGGATCTCAGCTTC
shRNA		
Control	TTCTCCGAACGTGTCACGT	
sh*CDC20*-1	GCACTGGACAACAGTGTGTAC	
sh*CDC20*-2	GCAGAAACGGCTTCGAAATAT	

### Oil red O staining

BMSCs and hASCs were cultured in PM and AM, separately. At day 21, Oil red O staining was conducted. In brief, after flushing with phosphate-buffered saline (PBS), cells were fixed with 10% formalin. The cells were then rinsed with 60% isopropyl alcohol for 10 min and incubated with 0.3% Oil red O. After 100% isopropyl alcohol was added to the stained cells, quantitative measurement was conducted by determining their spectrophotometric absorbance at 520 nm.

### RNA isolation and qRT-PCR

RNA was obtained using the TRIzol reagent (Invitrogen, Waltham, MA, USA). The RNA was reversed transcribed into cDNA using a PrimeScript RT Reagent Kit (Takara, Dalian, China). The cDNA was used as the template for the qPCR step of the qRT-PCR protocol using SYBR Green Master Mix (Roche Applied Science, Branchburg, NJ, USA). The expression of glyceraldehyde 3-phosphate dehydrogenase (GAPDH) was used for normalization of qRT-PCR data. The 2^−ΔΔCt^ method was used to analyze the relative gene expressions. The primers for indicated genes are shown in Table 2.

### Western blotting analysis

Cells were collected, washed with PBS and lysed using radioimmunoprecipitation assay (RIPA) lysis buffer with 2% protease inhibitor for 30 min. After centrifugation at 12,000 × *g* for 20 min, the isolated proteins were subjected to 10% or 15% SDS-PAGE and transferred to the PVDF membranes. After blocking in 5% dehydrated milk for 1–3 h, membranes were then incubated with primary antibodies against PPARγ (Abcam, Cambridge, MA, USA; 178,860), C/EBPα (Proteintech, Rosemont, IL, USA; 18,311–1-AP), fatty acid binding protein 4 (FABP4) (Proteintech; 12,802–1-AP), CDC20 (Abcam; ab183479), and β-catenin (Proteintech; 51,067–2-AP) overnight. Membranes were washed with PBS, incubated with the secondary antibody solution for 1 h, and then washed with PBS for three times. The immunoreactive protein bands were observed using an enhanced chemiluminescence (ECL) kit (CWBIO, Beijing, China). The protein levels were quantified through ImageJ software (National Institutes of Health, Bethesda, Maryland). GAPDH was used as internal control.

### In vivo adipose tissue formation

BALB/c nude mice were purchased from the Vital River Corporation (Beijing, China). BMSCs were induced in AM for 1 week and then mixed with a Collagen Sponge for 2 h. The mixture was then transplanted under the subcutaneous space of the nude mice and then collected 6 weeks later for histological observation.

### Immunochemistry

For immunofluorescence of bone sections, the fixed bone sections were blocked in 0.8% bovine serum albumin (BSA) for 1 h. Sections were incubated with the primary antibodies (1:200) against CDC20 (Abcam; ab183479) overnight, followed by incubation in 1:500 secondary antibody (ZF-0311, ZSGB-BIO, Beijing, China) for 1 h. Nuclei were stained using 4 ′,6-diamidino-2-phenylindole (DAPI).

### Histology and staining

The femurs and tibiae of mice were fixed in phosphate-buffered formalin for subsequent experiments. They were decalcified in 0.5 M EDTA for 2 weeks and then divided into two parts. One part was prepared for Oil red O staining. The other part was dehydrated and embedded in paraffin for hematoxylin and eosin (H&E) staining.

## Statistical analysis

Data are shown given as the mean ± standard deviation (SD). GraphPad Prism software (GraphPad Inc., San Diego, CA, USA) was utilized. Independent two-tailed Student’s *t-*tests, one-way analysis of variance (ANOVA), and a Tukey’s post hoc test were utilized to examine the level of significance. *P* values less than 0.05 were considered statistically significant.

## Results

### Downregulation of *CDC20* in the process of adipogenic differentiation of hBMSCs.

**Fig. 1:**
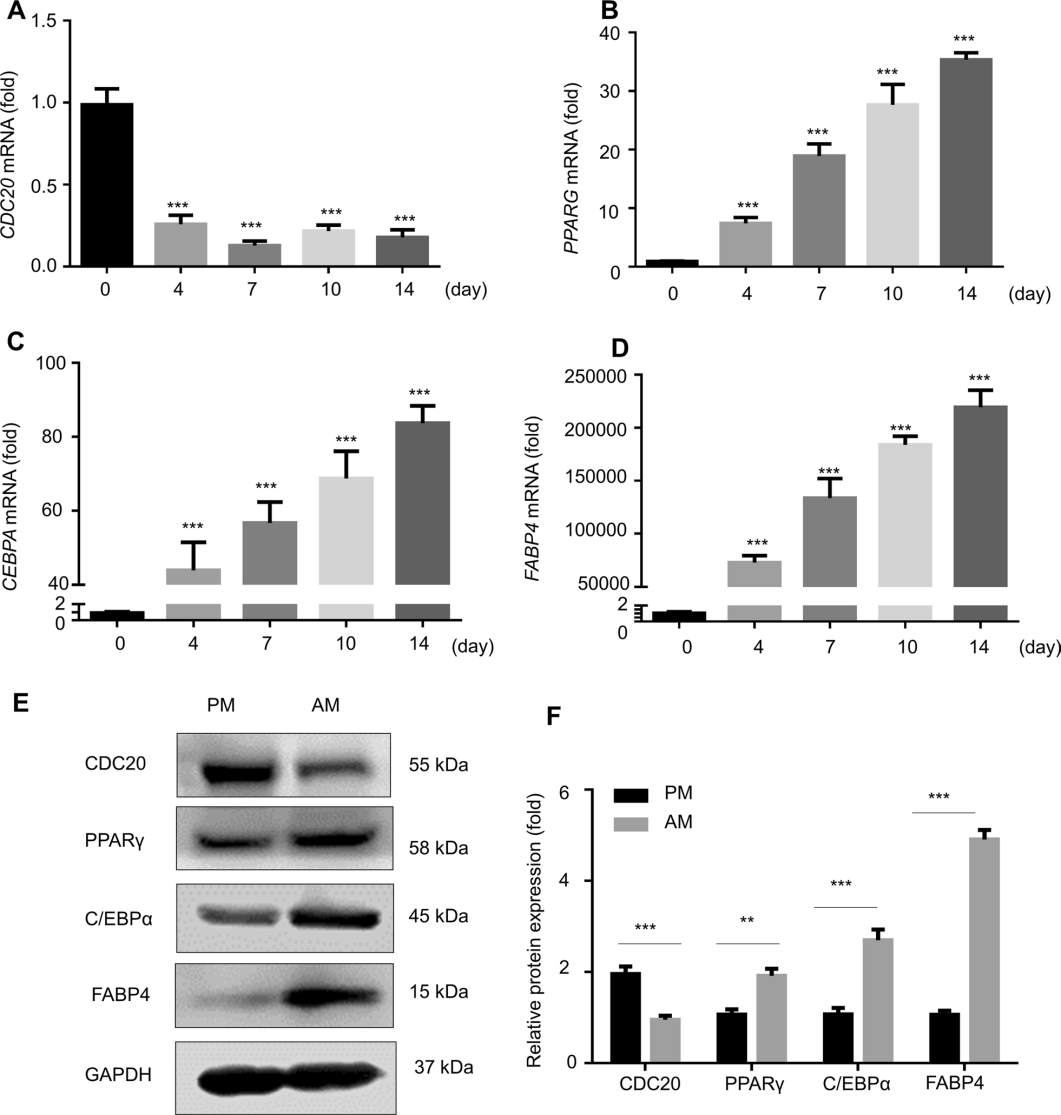
Downregulation of *CDC20* in the process of adipogenic differentiation of hBMSCs. **A** *CDC20* mRNA expression at day 0, 4, 7, 10, and 14 during the process of adipogenic differentiation of hBMSCs examined by qRT-PCR. (B-D) *PPARG* **B**, *CEBPA* **C**, and *FABP4* **D** mRNA expression levels at day 0, 4, 7, 10, and 14 during the process of adipogenic differentiation of hBMSCs examined by qRT-PCR. (E, F) Western blotting analysis **E** and quantification **F** of CDC20, PPAR*γ*, C/EBP*α*, FABP4 and the internal control GAPDH protein levels in proliferation medium (PM) and adipogenic medium (AM) for 7 days. All data are presented as the mean ± SD (*n* = 3, **P < 0.01, ***P < 0.001)

To determine whether CDC20 participates in BMSC adipogenesis, we assessed its mRNA expression during the process of adipogenic induction. The qRT-PCR results revealed that *CDC20* mRNA expression decreased significantly during adipogenic induction at day 0, 4, 7, 10, and 14 (Fig. 1A). Meanwhile, expression of the adipocyte regulator genes *PPARG*, *CEBPA*, and *FABP4* increased during adipogenic induction (Fig. 1B-D). Accordingly, western blotting analysis showed that adipocyte regulators PPARγ, C/EBPα, and FABP4 protein levels were upregulated, while CDC20 levels decreased during adipogenic induction of hBMSCs for 7 days (Fig. 1E, F).

### Knockdown of *CDC20* enhances adipogenic differentiation of hBMSCs

**Fig. 2:**
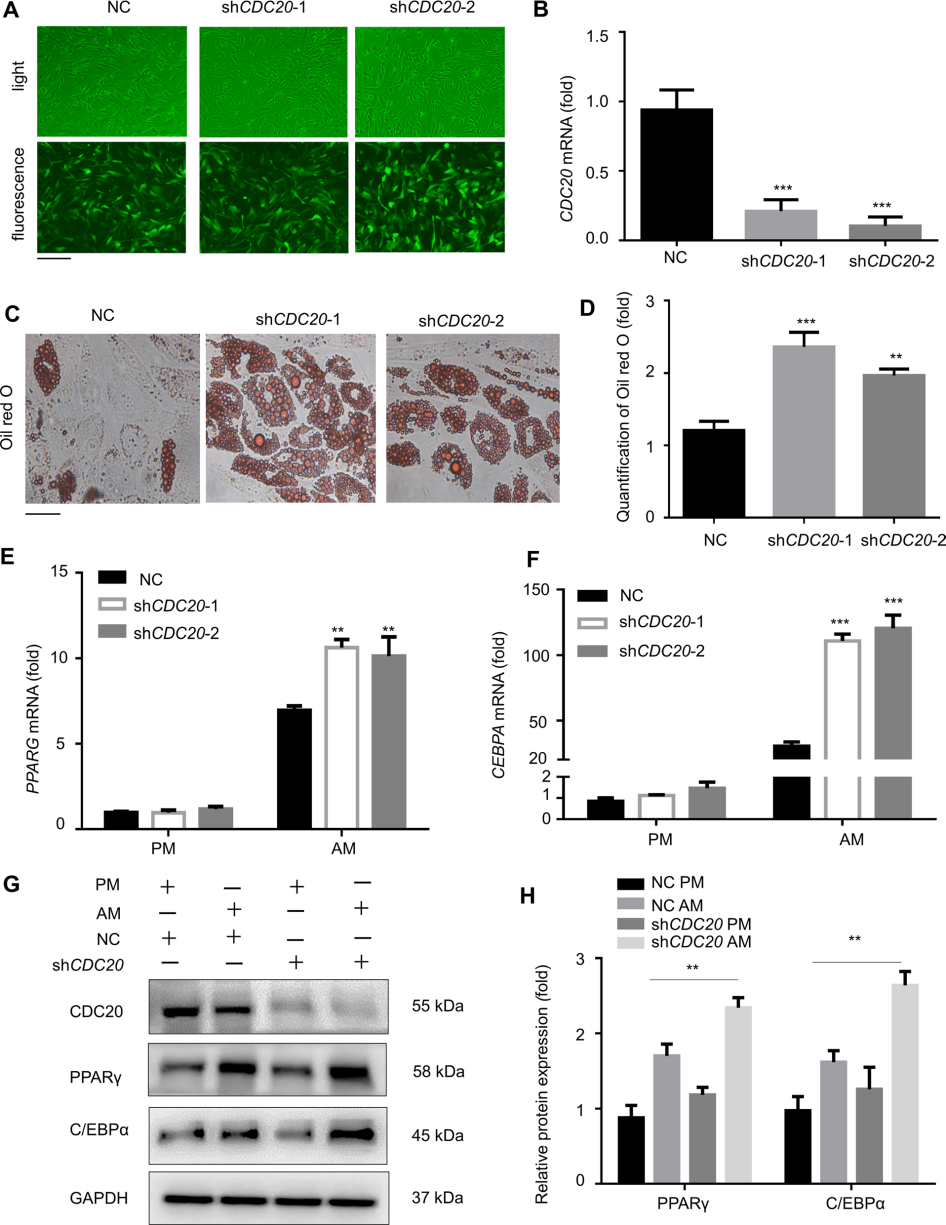
Knockdown of *CDC20* enhances adipogenic differentiation of hBMSCs. **A** Fluorescence micrographs showing the lentivirus transduction efficiency. Scale bar, 500 μm. **B** *CDC20* mRNA expression in control shRNA (NC) and *CDC20* knockdown (sh*CDC20*-1, sh*CDC20*-2) groups examined using qRT-PCR. (C, D) Oil red O staining **C** and quantification **D** of hBMSCs after adipogenic induction for 21 days. Scale bar, 100 μm. (E, F) Adipogenic regulators *PPARG* **E** and *CEBPA* **F** mRNA expression levels determined by qRT-PCR after adipogenic induction for 14 days. (**G**, **H**) Western blotting analysis **G** and quantification **H** of CDC20, PPARγ, C/EBPα, and the internal control GAPDH of negative control (NC) and *CDC20* knockdown (sh*CDC20*) cells in proliferation medium (PM) and adipogenic medium (AM) for 7 days. All data are presented as the mean ± SD (*n* = 3, **P < 0.01, ***P < 0.001)

To investigate the function of CDC20 in adipogenic differentiation, we constructed *CDC20* stable knockdown hBMSCs. To assess the effect of CDC20 in other cells lines, we performed the experiments in human adipose-derived stem cells (hASCs). The *CDC20* shRNAs and controls were transfected into hBMSCs and hASCs. Fluorescence staining showed that the lentiviral transduction efficiency was > 90% (Fig. 2A, Additional file 1: SFig 1A). Similarly, the qRT-PCR results showed that *CDC20* expression decreased dramatically after transfection (Fig. 2B, Additional file 1: SFig 1B). After cultivating hBMSCs and hASCs in AM for 21 days, *CDC20* knockdown notably increased adipogenesis, according to Oil red O staining (Fig. 2C, D; Additional file 1: SFig 1C, D). *PPARG* and *CEBPA* mRNA expression increased significantly after adipogenesis was induced by *CDC20* knockdown in hBMSCs (Fig. 2E, F). Western blotting revealed that PPARγ and C/EBPα protein levels increased after *CDC20* silencing in adipogenic medium for 7 days (Fig. 2G, H).

### Knockdown of *CDC20* promotes the formation of newly generated adipose tissue of hBMSCs

**Fig. 3:**
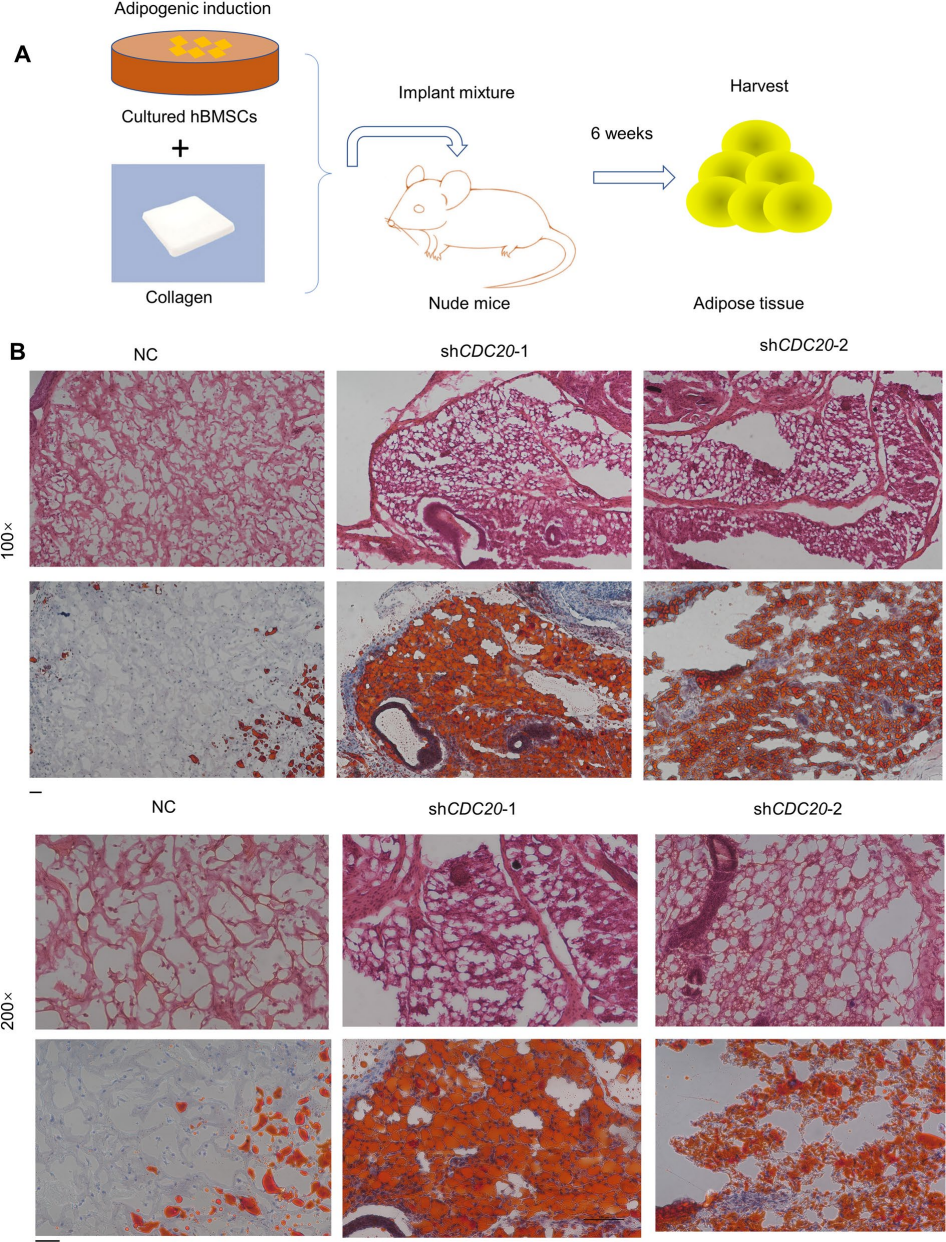
Knockdown of *CDC20* promotes the formation of newly generated adipose tissue of hBMSCs. **A** Schematic diagram demonstrating the procedure. **B** Oil red O staining and H&E staining in the NC, sh*CDC20*-1 and sh*CDC20*-2 groups. Scale bar, 50 μm; *n* = 3

To further examine the adipogenic role of CDC20 in hBMSCs, we implanted hBMSCs with stable knockdown of *CDC20* (sh*CDC20*-1, sh*CDC20*-2), or the negative control (NC), with a collagen scaffold, into nude mice. The newly generated tissue was gathered 6 weeks later. A diagram of this process is shown in Fig. 3A. The adipose tissue was further assessed by H&E staining and Oil red O staining, which revealed intracellular lipid accumulation. The sh*CDC20* groups generated more adipose-tissue-like positively stained complexes, while the NC group possessed fewer lipid droplets, as revealed by H&E and Oil red O staining (Fig. 3B).

### Conditional knockout of *Cdc2*0 promotes adipogenesis in the bone marrow of mice

**Fig. 4:**
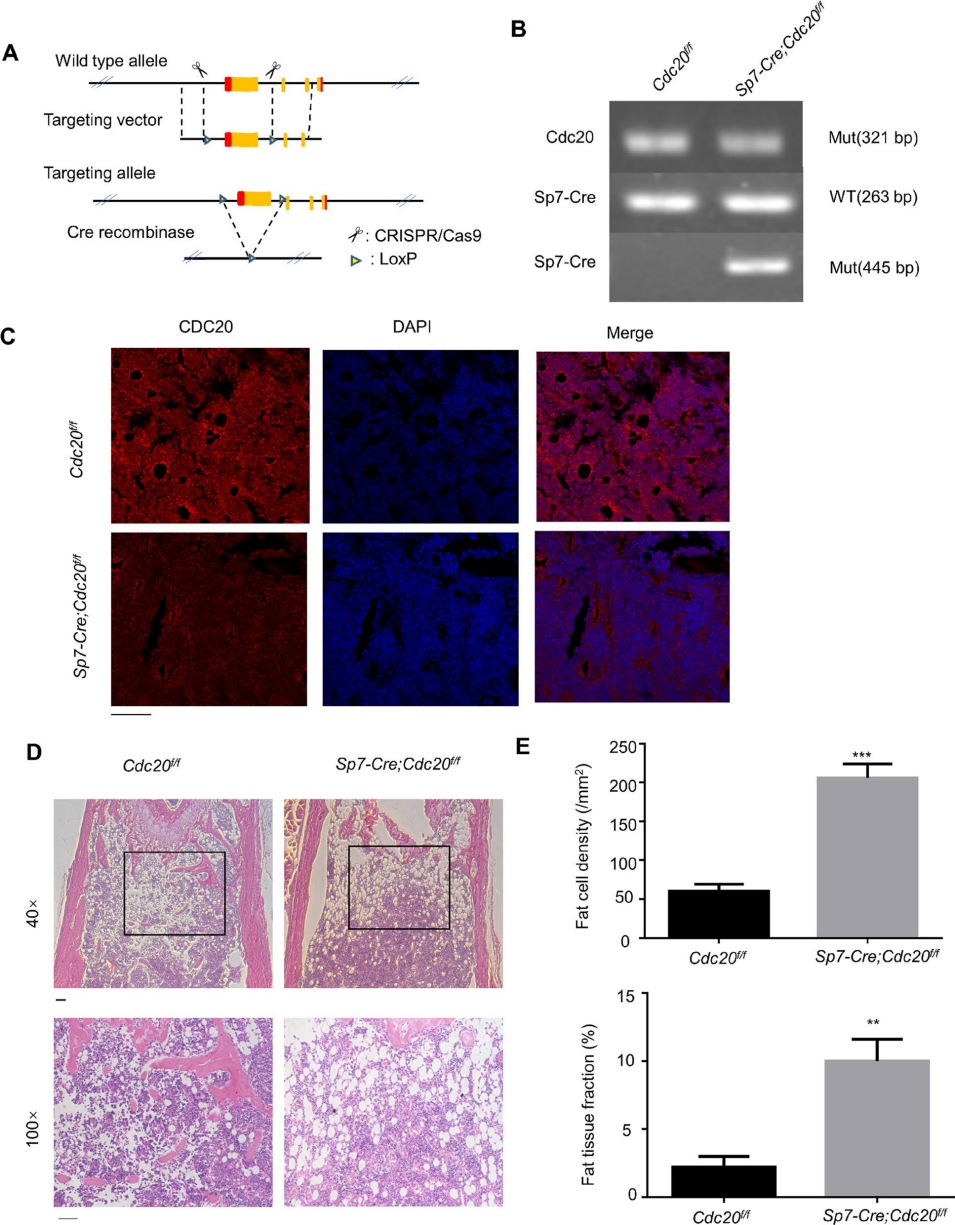
Conditional knockout of *Cdc20* promotes adipogenesis in the bone marrow of mice. **A** The design of the conditional deletion of th*e Cdc20* gene. **B** Images of PCR genotypes of control (*Cdc20*^*f/f*^) and conditional knockout (*Sp7-Cre;Cdc20*^*f/f*^) mice. **C** Immunofluorescence of the relative expression of CDC20 in bone sections of control (*Cdc20*^*f/f*^) and conditional knockout (*Sp7-Cre;Cdc20*^*f/f*^) mice. Scale bar, 100 μm. **D** H&E staining of paraffin sections from femurs and tibiae of 6-week-old control (*Cdc20*^*f/f*^) and conditional knockout (*Sp7-Cre;Cdc20*^*f/f*^) mice. Scale bar, 100 μm. **E** Quantification of fat cell density and fat tissue fraction based on the H&E staining of *Cdc20*^*f/f*^ and *Sp7-Cre;Cdc20*^*f/f*^ mice. All data are presented as the mean ± SD (*n* = 3, **P < 0.01, ***P < 0.001)

Next, we explored the effect of CDC20 on adipogenesis in vivo. We deleted *Cdc2*0 specifically from skeletal tissue in our mouse model (Fig. 4A). The genotypes of constructed mice were examined (Fig. 4B). *Cdc20*^*f/f*^ were the control mice, and *Sp7-Cre;Cdc20*^*f/f*^ were the conditional knockout mice. In the femur sections of the mice, the *Cdc20* knockout efficiency in bone was measured using immunofluorescence (Fig. 4C). H&E staining of the femoral and tibial metaphysis of 6-week-old *Sp7-Cre;Cdc20*^*f/f*^ mice presented increased numbers of adipocytes in the bone marrow compared with those in *Cdc20*^*f/f*^ mice (Fig. 4D). The adipocytes were measured using ImageJ software, which determined the fat cell density as well as the fat tissue fraction. Dramatic differences were observed between *Cdc20*^*f/f*^ and *Sp7-Cre;Cdc20*^*f/f*^ mice (Fig. 4E), demonstrating that conditional knockout of *Cdc20* promoted the differentiation of mesenchymal stem cell and osteoprogenitors into adipocytes.

### Conditional knockout of *Cdc20* increases adipogenic differentiation of mBMSCs

**Fig. 5:**
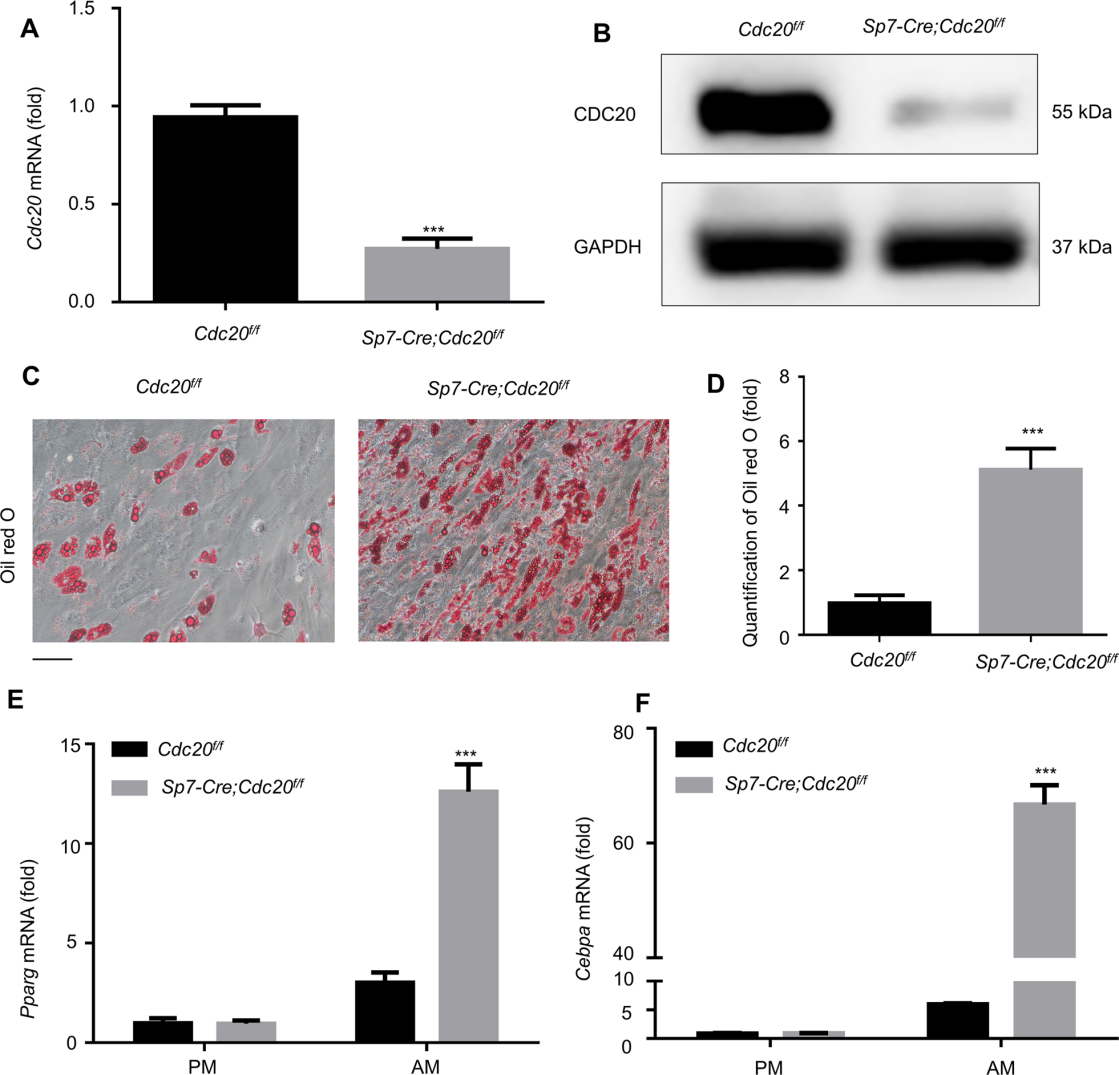
Conditional knockout of *Cdc20* increases adipogenic differentiation of mBMSCs. (**A**, **B**) The knockout efficiency of *Cdc20* in BMSCs of *Cdc20*^*f/f*^ and *Sp7-Cre;Cdc20*^*f/f*^ mice examined by qRT-PCR **A** and western blotting analysis **B**. (C, D) Oil red O staining **C** and quantification **D** at day 21 after adipogenic induction of mBMSCs from *Cdc20*^*f/f*^ and *Sp7-Cre;Cdc20*^*f/f*^ mice. Scale bar, 100 μm. (**E**, **F**) Adipogenic markers *Pparg* **E** and *Cebpa* **F** mRNA expressions determined by *q*RT-PCR after adipogenic induction for 14 days of mBMSCs from *Cdc20*^*f/f*^ and *Sp7-Cre;Cdc20*^*f/f*^ mice. All data are presented as the mean ± SD (*n* = 3, ***P < 0.001)

The mBMSCs were flushed out of mouse long bones and collected. The qRT-PCR results (Fig. 5A) and western blotting analysis (Fig. 5B) determined the knockdown efficiency. After adipogenic induction for 21 days, *Cdc20* deletion in mBMSCs significantly enhanced adipogenic differentiation, as determined by Oil red O staining and quantification (Fig. 5C, D). *Pparg* and *Cebpa* mRNA expression levels increased significantly following *Cdc20* deletion in mBMSCs (Fig. 5E, F), which demonstrated that conditional knockout of *Cdc2*0 increased mBMSC adipogenic differentiation.

### Knockdown of *CDC20* decreases *β*-catenin expression

**Fig. 6:**
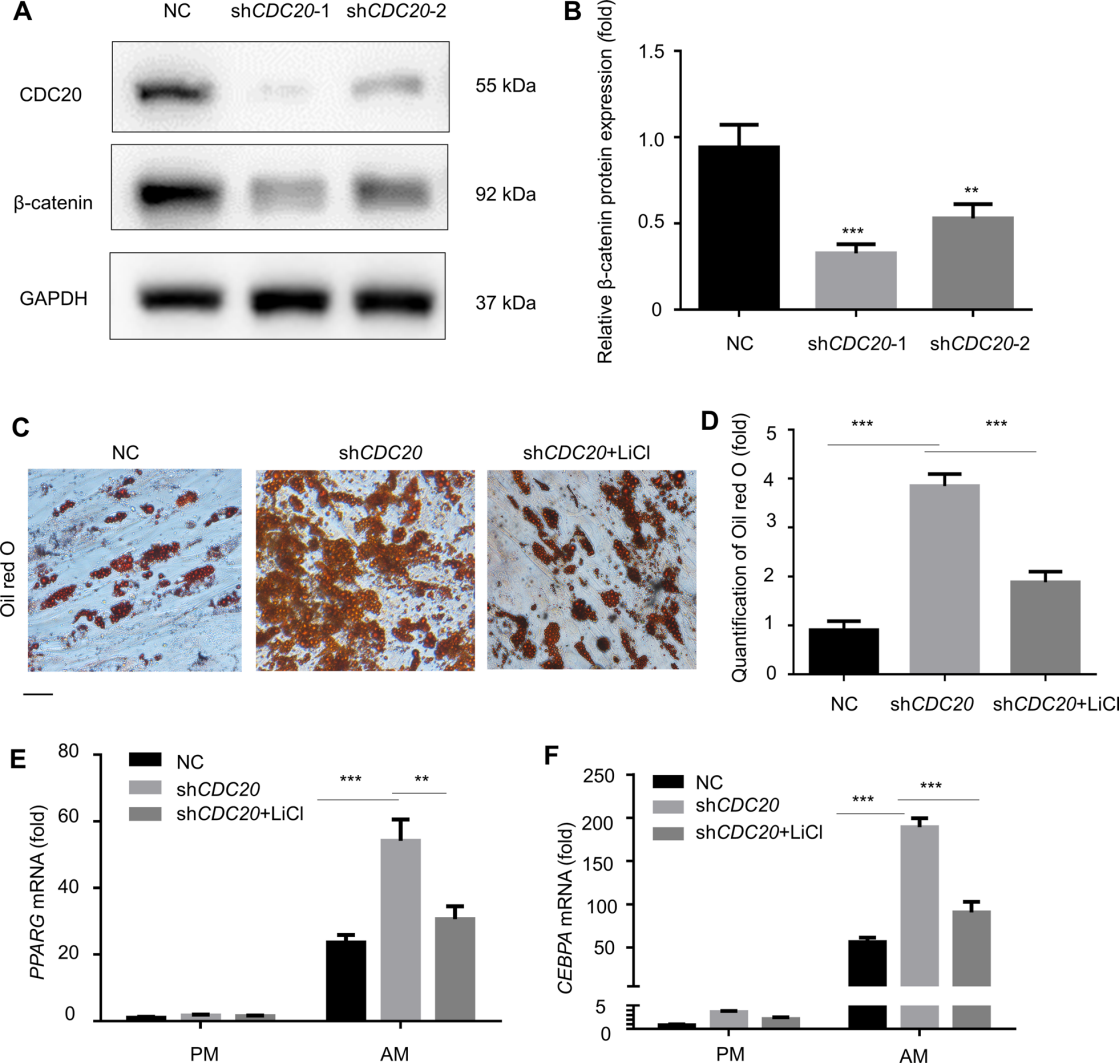
Knockdown of *CDC20* decreases β-catenin expression. (**A**, **B**) The level of *β*-catenin decreased in *CDC20* knockdown cells, as determined by western blotting analysis **A** and quantification **B**. (**C**, **D**) Oil Red O staining **C** and quantification **D** after treatment with an activator of Wnt/*β*-catenin signaling (LiCl, 5 mmol/L) for 21 days in *CDC20* knockdown cells. Scale bar, 100 μm. (**E**, **F**) The mRNA expression of *PPARG* **E** and *CEBPA* **F** after treatment with LiCl (5 mmol/L) for 14 days in *CDC20* knockdown cells. All data are presented as the mean ± SD (*n* = 3, **P < 0.01, ***P < 0.001)

To investigate the underlying molecular mechanisms of CDC20’s effect on the adipogenic differentiation of BMSCs, we examined signaling pathways related to adipogenesis. Interestingly, we found that the protein level of β-catenin decreased after knockdown of *CDC20* according to western blotting (Fig. 6A, B). Then, we activated Wnt/β-catenin signaling by adding LiCl. After adipogenic induction, Oil red O staining showed that LiCl treatment (5 mmol/L) decreased adipogenesis caused by knockdown of *CDC20* (Fig. 6C, D). Similarly, LiCl reduced the *PPARG* and *CEBPA* mRNA expression in *CDC20* knockdown BMSCs (Fig. 6E, F).

## Discussion

The imbalance between bone formation and resorption might contribute to various diseases, such as osteoporosis, osteopenia, and osteopetrosis [1]. Studies have demonstrated that the regulation of bone and fat mass are closely connected [23, 24]. The bone and fat in bone marrow were thought to show an inverse relationship. The anti-diabetic treatment, glitazone, was accompanied by bone loss, as well as increased bone marrow fat content [25]. Conditions with high bone mass resulting from mutant lipoprotein related receptor 5 (Lrp5) were accompanied by a decrease in bone mass [26]. Our previous studies have revealed that CDC20 positively regulates BMSC osteogenesis [21], and in this study, we demonstrated its negative effect on adipogenesis.

APC/C^CDC20^ recognizes the KEN box or D box of substrates to induce ubiquitination degradation, triggering the transition from metaphase to anaphase [27]. Studies have illustrated the effect of CDC20 in hematopoietic stem cells [28]. It is expressed in the layers of the epidermis, and regulates the fate of adult stem cells [29]. In our study, the deletion of *Cdc20* reprogrammed the fate determination of osteoprogenitors and BMSCs, causing a shift from osteoblasts to adipocytes, which might partly lead to enhanced bone marrow adiposity accompanied by low bone mass in the conditional knockout mice.

Several factors and intercellular pathways have been claimed to control the fate of BMSCs [30]. Accumulating information has identified that adipose tissue can secrete various active molecules, including adiponectin, interleukin 6 (IL-6), and leptin [31]. Bones secrete various active cytokines, such as osteoprotegerin, osteocalcin, and osteopontin [32]. These cytokines have reciprocal roles in bone and fat metabolism. As one of the major regulators of adipogenesis, Wnt/*β*-catenin plays essential roles in cell differentiation, migration, and gene expression [33]. The connection between *β*-catenin and CDC20 has been studied from many aspects. Silencing of *CDC20* inhibited the growth of prostate cancer by decreasing β-catenin levels [34, 35]. Knockdown of *CDC20* inhibited Wnt/*β*-catenin signaling through conducting during cell cycle [36]. In addition, knockdown of *CDC20* increased cell apoptosis and decreased their migratory ability by inhibition of the Wnt/β-catenin pathway [37]. In this study, we demonstrated that CDC20 inhibited the adipogenic differentiation of BMSCs by regulating *β*-catenin, which provides evidence for the manipulation of bone marrow fat.

Using conditional knockout mice constructed through the Cre/LoxP system driven by *Sp7-Cre* [38], our lineage tracing showed that *Cdc20* deletion from osteoprogenitors induced them to become adipocytes in the bone marrow microenvironment. Other researchers used *Osx* (Osterix, also known as Sp7)*-Cre* mice to investigate the lineage commitment of mesenchymal stem cells. *Cbfβ*^*f/f*^*Osx-Cre* mice showed serious osteoporosis with a significant increase in adiposity [39]. Mice with an osteoprogenitor-specific *Dkk1* (encoding Dickkopf WNT signaling pathway inhibitor 1) deletion presented an accumulation of bone marrow fat and a protected against cortical bone loss induced by a high-fat diet [40]. Using *Osx-Cre*^*ERT2*^ mice, TSC complex subunit 2 (TSC2) haploinsufficiency in osteoprogenitors attenuated the increase in bone marrow fat [41].

However, there are several limitations of our study. The underlying mechanisms of how CDC20 regulates adipogenic differentiation and the balance of bone-fat turnover were not fully determined. Additionally, whether CDC20 influences whole body fat accumulation requires examination in following studies, which would be significant in clinical applications.

## Conclusion

Overall, our study showed that *CDC20* knockdown increased adipogenic differentiation of BMSCs by decreasing β-catenin levels and induced adiposity accumulation in bone marrow. Furthermore, CDC20 might be involved in balancing adipogenesis and osteogenesis in stem cells in tissue engineering and as a therapeutic target in the treatment of osteoporosis.

## Supplementary Information

**Additional file1: Fig. S1.** Knockdown of CDC20 enhances adipogenic differentiation of hASCs. (**A**) Fluorescence micrographs showing the lentivirus transduction efficiency. Scale bar, 500 μm. (**B**) Relative mRNA expression of CDC20 in control shRNA (NC) and CDC20 knockdown (shCDC20-1, shCDC20-2) hASCs examined by qRT-PCR. (**C**, **D**) Oil red O staining (**C**) and quantification (**D**) of hASCs after adipogenic induction for 21 days. Scale bar, 100 μm. All data are presented as the mean ± SD (*n*=3, ***P < 0.001).

## Data Availability

All data generated or analyzed during this study are included in this published article.

## Abbreviations

AM: Adipogenic medium
*α*-MEM: *α*-Minimum essential medium
APC/C: Anaphase-promoting complex/cyclosome
BMSCs: Bone marrow-derived mesenchymal stromal/stem cells
CDC20: Cell division cycle 20
DMEM: Dulbecco’s modified eagle’s medium
FBS: Fetal bovine serum
hASCs: Human adipose-derived stem cells
H&E: Hematoxylin and eosin
LiCl: Lithium chloride
NC: Negative control
Osx: Osterix
PM: Proliferation medium
