# Supplementary figures and images for: Knockdown of CDC20 promotes adipogenesis of bone marrow-derived stem cells by modulating β-catenin
Source: Stem Cell Res Ther. 2022 Sep 2;13:443. doi: 10.1186/s13287-022-03062-0 (PMC9438178; doi:10.1186/s13287-022-03062-0)

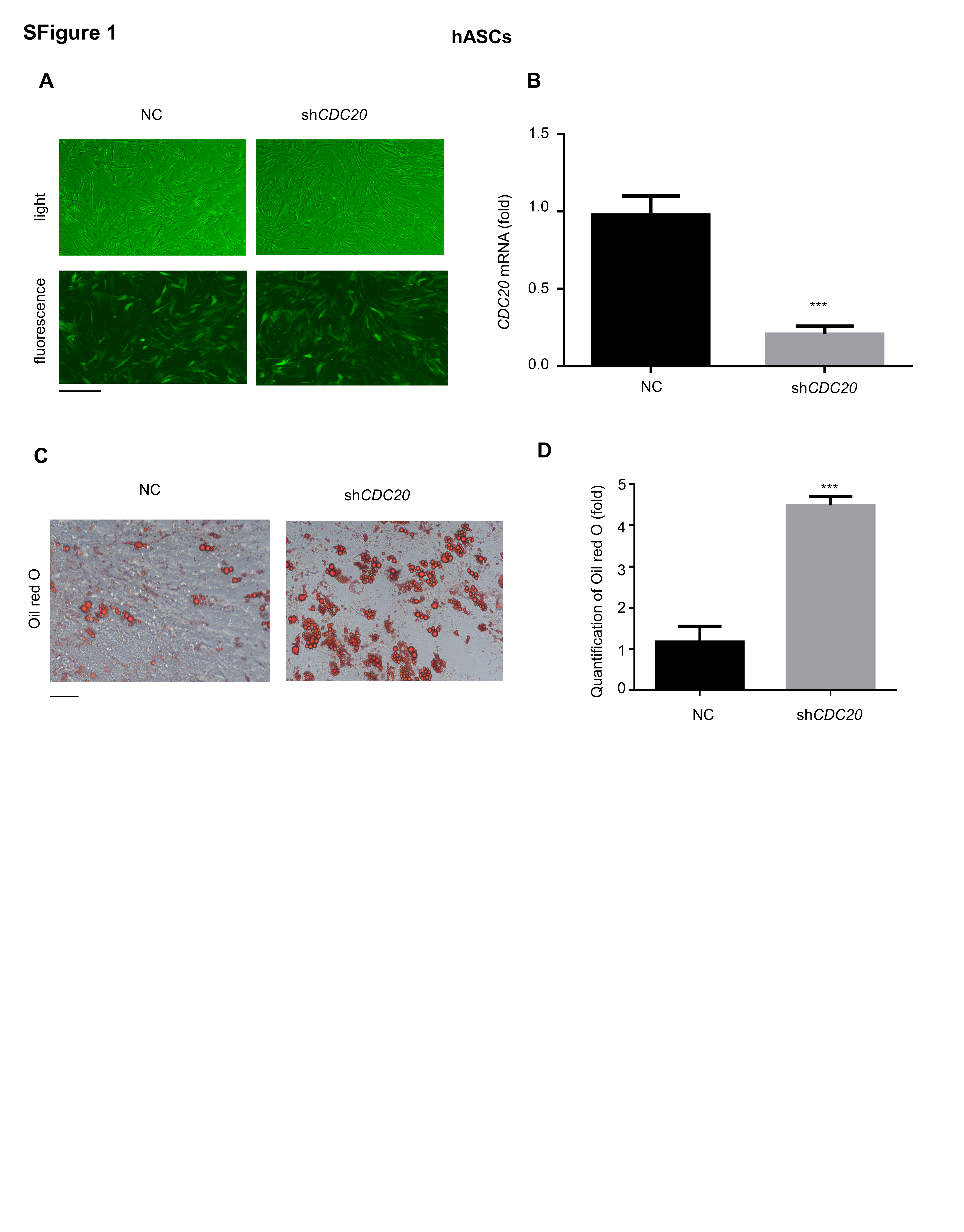

Supplement: Supplementary file 1 — Additional file1: Fig. S1. Knockdown of CDC20 enhances adipogenic differentiation of hASCs. (A) Fluorescence micrographs showing the lentivirus transduction efficiency. Scale bar, 500 μm. (B) Relative mRNA expression of CDC20 in control shRNA (NC) and CDC20 knockdown (shCDC20-1, shCDC20-2) hASCs examined by qRT-PCR. (C, D) Oil red O staining (C) and quantification (D) of hASCs after adipogenic induction for 21 days. Scale bar, 100 μm. All data are presented as the mean ± SD (n=3, ***P < 0.001). [file 13287_2022_3062_MOESM1_ESM.tif]
